# Butyrate inhibits the malignant biological behaviors of breast cancer cells by facilitating cuproptosis-associated gene expression

**DOI:** 10.1007/s00432-024-05807-1

**Published:** 2024-06-04

**Authors:** Liming Zhang, Shan Huang, Ying Yuan

**Affiliations:** 1 Jiangxi Provincial Key Laboratory of Medicine, Clinical Laboratory of the Second Affiliated Hospital of Nanchang University, No. 1, Minde Road, Donghu District, Nanchang, 330006 Jiangxi People’s Republic of China; 2 Neonatal Room, The Second Affiliated Hospital, Jiangxi Medical College, Nanchang University, Nanchang, 330008 Jiangxi People’s Republic of China; 3 Intensive Care Unit, The Second Affiliated Hospital, Jiangxi Medical College, Nanchang University, Nanchang, 330008 Jiangxi People’s Republic of China

**Keywords:** Breast cancer, Short-chain fatty acids, Cuproptosis, Cell migration, Cell invasion

## Abstract

**Background:**

Butyrate is a common short-chain fatty acids (SCFA), and it has been demonstrated to regulate the development of breast cancer (BC), while the underlying mechanism is still unreported.

**Methods:**

Gas chromatography was used to measure the amounts of SCFA (acetate, propionate, and butyrate) in the feces. Cell viability was measured by the CCK-8 assay. The wound healing assay demonstrated cell migration, and the transwell assay demonstrated cell invasion. The levels of protein and gene were determined by western blot assay and RT-qPCR assay, respectively.

**Results:**

The levels of SCFA were lower in the faecal samples from BC patients compared to control samples. In cellular experiments, butyrate significantly suppressed the cell viability, migration and invasion of T47D in a dose-dependent manner. In animal experiments, butyrate effectively impeded the growth of BC tumors. Toll like receptor 4 (TLR4) was highly expressed in the tumors from BC patients. Butyrate inhibited the expression of TLR4. In addition, butyrate promoted the expression of cuproptosis-related genes including PDXK (pyridoxal kinase) and SLC25A28 (solute carrier family 25 member 28), which was lowly expressed in BC tumors. Importantly, overexpression of TLR4 can reverses the promotion of butyrate to PDXK and SLC25A28 expression and the prevention of butyrate to the malignant biological behaviors of T47D cells.

**Conclusion:**

In summary, butyrate inhibits the development of BC by facilitating the expression of PDXK and SLC25A28 through inhibition of TLR4. Our investigation first identified a connection among butyrate, TLR4 and cuproptosis-related genes in BC progression. These findings may provide novel target for the treatment of BC.

**Supplementary Information:**

The online version contains supplementary material available at 10.1007/s00432-024-05807-1.

## Introduction

Breast cancer (BC) is one of the leading causes of cancer-related death, accounting for 15% of female deaths in 2018 and 25% of cancers in women globally (Xu et al. 2020; Bray et al. 2018). BC is characterized by high morbidity, high mortality, and high recurrence rate. Over the past two decades, the approach to the treatment of BC have evolved, incorporating various modalities such as surgery, chemotherapy, radiation therapy, targeted therapeutics, and other new emerging technologies (Lau 2022; Kerr et al. 2022). Neoadjuvant chemotherapy has shown promise in personalized treatment of BC, but its effectiveness varies among individuals and can lead to drug resistance (Korde and Somerfield 2021; Mao et al. 2021; Sheikh et al. 2022). Therefore, a deeper understanding of the pathogenic factors and molecular mechanisms involved in the pathogenesis of BC is necessary.

Mounting evidence has demonstrated the significant role of gut microbiota in cancer development, acting as a potential preventive or risk factor for cancers through various mechanisms such as affecting immune response, drug resistance and other events (Long et al. 2023; Fernandes et al. 2022). Clinical trial data has revealed significantly differences in gut microbiota proportion in fecal sample of the patients with BC, such as *Faecalibacterium prausnitzii*, *Bifidobacterium*, *Akkermansia muciniphila* and *Blautia* (Laborda-Illanes et al. 2020; Luu et al. 2017; Fuhrman et al. 2014). The metabolites released by gut microbiota, such as bile acids, short-chain fatty acids (SCFA) and branched-chain fatty acids (BCFAs), can impact the migration, invasion, proliferation and apoptosis of cancer cells (Hou et al. 2022). Commonly, SCFAs like propionate, acetate, and butyrate have been proved to exhibit anti-cancer and anti-inflammation properties (Nakkarach and Foo 2021; González-Bosch et al. 2023). For instance, Park et al. (2021) have indicated that propionate suppressed the proliferation and contributed the apoptosis of BC cells, and impedes the growth of tumor in nude mice by regulating STAT3/MAPK signalling pathway. Butyrate has been reported to have anti-cancer effect in the development of BC, and it can induce apoptosis of BC cells by affecting the formation of reaction oxygen species and impairing mitochondria (Sharma and Tollefsbol 2022; Salimi et al. 2017), but the related mechanism is still unclear. Studies have indicated that toll like receptor 4 (TLR4) could be a downstream target of butyrate, mediating its anti-inflammatory effects (Liu et al. 2022). Activation of TLR4, especially upon binding with lipopolysaccharide, has been linked to promoting the progression of BC by inducing gene expression associated with cancer progression (Afroz et al. 2022).

Cuproptosis is a novel discovered Cu^2+^-dependent pathway of cell death, which is closely associated with the metabolism of mitochondria. It has been demonstrated that cuproptosis plays an important auxiliary function in the development of cancers, potentially by inhibiting metastasis and impeding cancer development (Tong et al. 2022). For instance, Ferredoxin 1, a key gene involved in cuproptosis, has been identified as a suppressor of clear cell renal cell carcinoma (Xie et al. 2022). The upregulation of cuproptosis-related gene solute carrier family 31 member 1 (SLC31A1) in BC tumors is associated with higher risk and shorter overall survival (Li et al. 2022a). While cuproptosis-related genes show promise as prognostic and therapeutic targets for BC, but the understanding of the functions of cuproptosis in the pathogenesis of BC is inadequate.

In the current study, we focused on the effects of butyrate on the cell viability, migration and invasion of BC cell and the growth of BC tumor. Meantime, we detected the regulation of butyrate to its downstream target TLR4 and the cuproptosis-related genes [pyridoxal kinase (PDXK) and solute carrier family 25 member 28 (SLC25A28)], so as to investigate the role and underlying mechanism of butyrate in BC.

## Materials and methods

### Analysis of SCFA concentration

Faecal samples were obtained from 12 patients with BC, whose underwent operative treatment in our hospital from June 2020 to October 2021. At the same time, 12 healthy volunteers were recruited for the collection of control faecal samples. The histological classifications of BC patients were listed in Supplementary Table 1. Every participant was provided with sterile plastic containers for the collection of faecal samples. Faecal SCFA (acetate, propionate and butyrate) level was measured by using gas chromatograph (Shimadzu Corporation, Kyoto, Japan) according to previous studies (Birkeland et al. 2020; Ubachs et al. 2021). Frozen faeces (500 mg) were dried in a vacuum dryer, and the level of SCFA was corrected for dry weight. The levels of acetate, propionate, and butyrate were presented as mM/g.

### Collection of clinical and cell samples

12 pairs of tumor tissue and adjacent paracancerous tissue were obtained from the female patients with BC. This study was approved by the ethical committee of The Second Affiliated Hospital, Jiangxi Medical College, Nanchang University. Written informed consents were received from all patients. Tumors were stored in − 80 °C until to use. The mRNA and protein levels of TLR4, PDXK and SLC25A28 in tumors and non-tumors were examined.

BC cell lines (T47D and MCF-7) and HEK293 (a human embryonic kidney cell line) were obtained from American Type Culture Collection (ATCC; USA). Cells were cultured in RPMI-1640 medium (Hyclone, Logan, UT, USA) supplemented with 1% penicillin–streptomycin (Invitrogen, Carlsbad, USA) and 10% fetal bovine serum (FBS; Invitrogen) in an incubator with 5% CO_2_. The ambient temperature during cell culture was 37 °C. T47D cells were treated with 0, 2.0 and 5.0 mM concentration of butyrate (Selleck Chemicals, Houston, USA) for 48 h. The plasmid expressing TLR4 and empty plasmid were purchased from Hanbio Biotechnology Co., Ltd. (Shanghai, China). Cell transfection was carried out by using Lipofectamine 3000 (Invitrogen) according the instruction.

### CCK-8 assay

The viability of T47D cells was assessed using a Cell Counting Kit-8 (Solarbio, Beijing, China). T47D cells were seeded into a 96-well plate a density of 2 × 10^3^ cells/well, then either treated with butyrate or transfected with the plasmid expressing TLR4. Then, 10 μL of CCK-8 solution was added to each well, and the cells were incubated for 1 h at 37 °C. The absorbance at 450 nm was measured using a microplate reader (BioTek ELx800, USA).

### Wound healing assay

T47D cells were seeded to 6-well plate at a density of 1 × 10^5^ cells/well. After 12 h of cell culture, the suspended cells were washed with 0.01 M PBS and the cell medium was changed. To draw a horizontal line in the bottom of the cell culture plate, a 200 μl pipette tip was held perpendicularly to the plate. The time was written down as 0 h at this point. After 48 h of butyrate treatment and the transfection of plasmid expressing TLR4, cell migration was measured using Image J software.

### Transwell assay

Serum-free medium was added into the upper chamber of Transwell (Corning Costar, USA), which was per-coated with matrigel (Matrigel GFR Membrane Matrix, Corning Costar). T47D cells were planted at the upper chamber at a density of 5 × 10^4^ cells/well. The cell culture medium supplemented with 20% FBS was added into the lower chamber. After 48 h of butyrate treatment and cell transfection, the cells attached to the bottom of upper chamber were fixed with 4% paraformaldehyde, and were then stained with crystal violet. The invasive cells were captured by using a microscope and the number of the cells was measured by Image J software.

### Western blot assay

The protein levels of TLR4, PDXK and SLC25A28 in tumors or BC cells were determined by western blot assay. Total protein was extracted from the experimental samples by using RIPA lysis buffer (Solarbio). After determining the concentration of protein by using a BCA Protein Assay Kit (Beijing Dingguochangsheng Biotechnology Company, LTD), 25 μg protein per sample was loaded onto 12% SDS-PAGE gels and separated. Then, proteins were transferred onto PVDF membrane (0.45 μm; Millipore, Carrigtwohill, Ireland), which was maintained with 5% non-fat milk for 1 h at room temperature. The membranes were incubated with primary antibodies including TLR4 (1:2000; Cell Signaling Technology, USA), PDXK (1:2000; Cell Signaling Technology) and SLC25A28 (1:2000; Cell Signaling Technology) overnight at 4 °C. After that, the membranes were incubated with HRP-conjugated goat anti-mouse or goat anti-rabbit secondary antibodies (Proteintech, Wuhan, China) for 1 h at room temperature. At last, protein bands were analyzed by using an ECL kit (Solarbio) and Image J software. GAPDH served as internal control.

### RT-qPCR assay

TRIzol reagent (Invitrogen) was utilized for extraction of total RNA, which was then reverse transcripted into cDNA by using HiScript III RT SuperMix kit (Vazyme Biotech, Nanjing, China). After that, qPCR was carried out to determine the expression levels of TLR4, PDXK and SLC25A28 mRNAs by using SYBR Green PCR Master Mix (TAKARA, Dalian, China). Gene expression was analyzed using the 2^−ΔΔCt^ method. *GAPDH* served as the reference gene. Primer sequences used in our study are listed as follows: GAPDH: forward, 5′-TGACTTCAACAGCGACACCCA-3′ and reverse, 5′-C ACCCTGTTGCTGTAGCCAAA-3′; TLR4: forward, 5′-GAGCCGGAAGGTTATTT GGT-3′ and reverse, 5′-CCTCTGCTGTTTGCTCAGGAT-3′; PDXK: forward, 5′-GTG TGGCTGGACTGTACTCT-3′ and reverse, 5′-GCACATAACCTGCTCTGCTC-3′; SLC25A28: forward, 5′-CTGCGTGATGTACCCCATCG-3′ and reverse, 5′-CCTGTT GCTGTGACGTTCAG-3′.

### Animal experiments

All animal experiments were supported by the Animal Care Committee of The Second Affiliated Hospital, Jiangxi Medical College, Nanchang University. Eighteen female BALC/c nude mice (weight at 15 ± 2 g, aged 4 ± 1 weeks) were purchased from the Charies river (Beijing, China). Mice were housed in SPF-grade animal room, and all of them have free access to water and food. Mice were subcutaneously implanted with slow-release 17β-estradiol pellets (0.25 mg). The following day, T47D cells were suspended in sterilized saline (1 × 10^6^ cells/100 μl) and injected subcutaneously into the left flank of nude mice. Then, tumor volume was determined every week for 4 weeks according to the method that volume (mm^3^) = width^2^ (mm^2^) × length (mm)/2. The next day after injection of T47D cells, experimental animals were randomly divided into three groups (n = 6 per group): 0 mg/kg group, 100 mg/kg group, and 200 mg/kg group. The mice in 100 mg/kg or 200 mg/kg group were administered intraperitoneally with 100 mg/kg or 200 mg/kg of butyrate (Selleck Chemicals, Houston, TX, USA) weekly for 4 weeks. The mice in 0 mg/kg were administrated with an equal volume of sterilized saline. At last, tumor was carefully removed from mice and frozen in liquid nitrogen immediately.

### Statistical analysis

Statistical analysis was accomplished using Graphpad Prism 8.0 (USA), and data were presented as mean ± standard deviation (SD). The difference between independent groups was determined by Student’s t-test, one-way analysis of variance followed by Tukey's post-hoc method was utilized for multiple groups. P < 0.05 was recognized as statistically significantly. * represents P < 0.05, ** means P < 0.01, and *** means P < 0.001.

## Results

### SCFA level was downregulated but TLR4 expression level was upregulated in BC

**Fig. 1:**
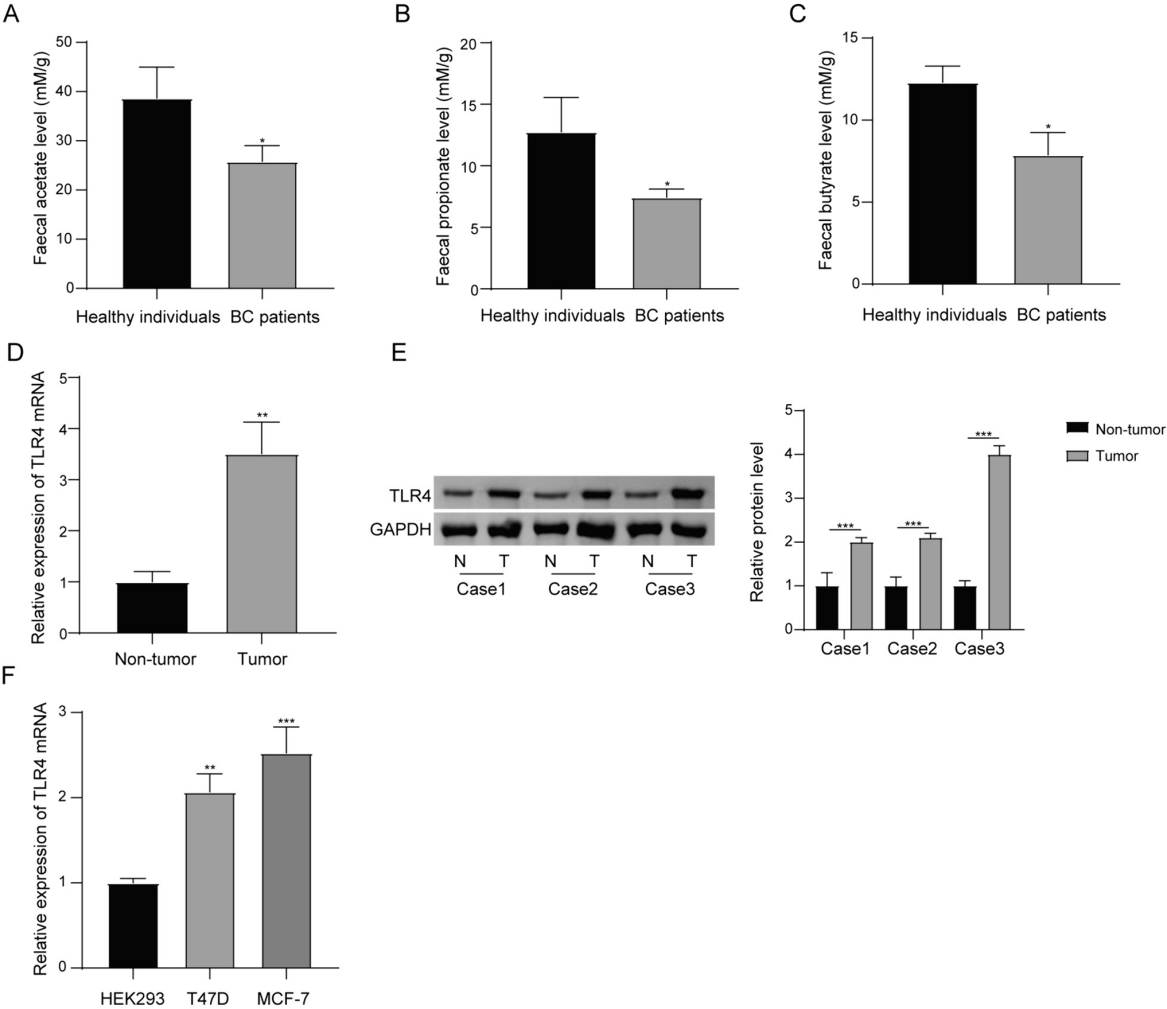
The levels of acetate, propionate and butyrate are decreased but the expression of TLR4 is increased in BC. **A, C** Faecal samples were obtained from the patients with BC or healthy individuals, and were used to measure the levels of acetate, propionate and butyrate by gas chromatography. **D** 12 cases of paracancerous tissue and 12 cases of BC tumor specimen were obtained and used for detecting the expression of TLR4 mRNA. **E** The expression levels of TLR protein in tumors and paracancerous tissues were determined by western blot assay, and three representative results were shown. **F** RT-qPCR revealed TLR4 mRNA expression in BC cell lines (T47D and MCF-7) and control cell line HEK293. *P < 0.05, **P < 0.01, and ***P < 0.001. N = 3

Studies have indicated that SCFA generated by gut microbiota is important for maintaining cell homeostasis and involves in the progression of BC(Mirzaei et al. 2021; Gonçalves et al. 2011). In the current study, the amounts of acetate (Fig. 1A), propionate (Fig. 1B) and butyrate (Fig. 1C) were lower in the feces samples of patients with BC when contrasted to healthy individuals. It has been demonstrated TLR4-mediated signalling pathways are crucial downstream targets of SCFA (Lu et al. 2022a). Here, higher TLR4 mRNA level was found in BC tumor when compared with paracancerous tissue (Fig. 1D). The expression of TLR4 protein was also more in BC tumor than that in paracancerous tissue (Fig. 1E). The expression levels of TLR4 mRNA in T47D and MCF-7 cell lines were higher than that in HEK293 cell line (Fig. 1F). Because of the above findings, we hypothesized that there may be some links between low SCFA level and high TLR4 level in the development of BC.

### Butyrate suppressed the malignant biological behaviors of T47D cells

**Fig. 2:**
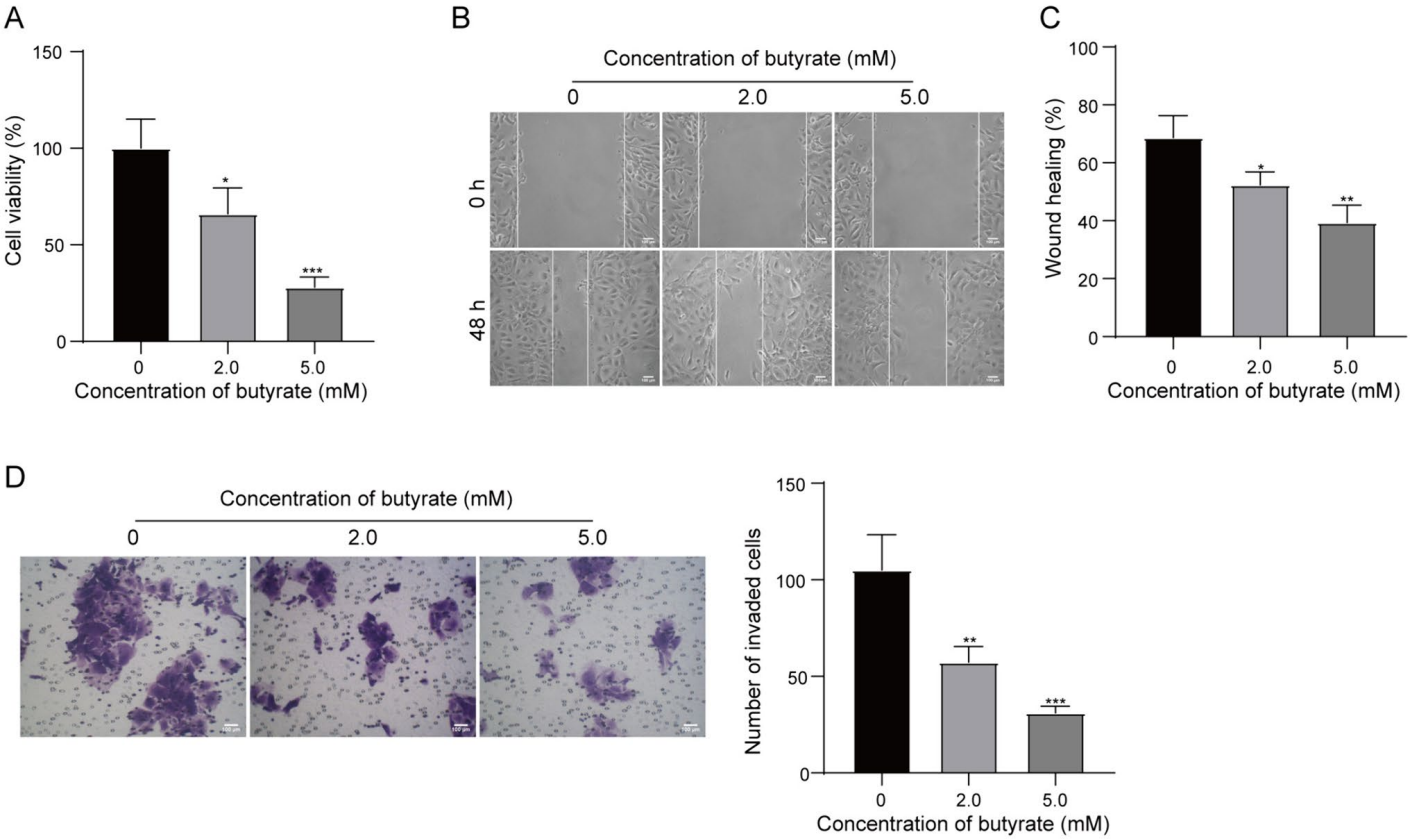
Butyrate inhibits BC cell viability, migration and invasion. T47D cells were treated with 0, 2.0 and 5.0 mM doses of butyrate. **A** Cell viability was determined by CCK-8 assay. **B** Wound healing assay was utilized for analyzing cell migration. (C) Transwell assay was used for detecting cell invasion. *P < 0.05, **P < 0.01, and ***P < 0.001. N = 3

In order to investigate the influence of SCFA on the malignant biological behaviors of BC cells, butyrate was selected as a representative SCFA in the present study. T47D cells were incubated with 0, 2.0 and 5.0 mM doses of butyrate. Then, cell viability was determined by using CCK-8 assay, and the results showed that butyrate significantly attenuated the cell viability of T47D in a dose-dependent manner (Fig. 2A). The migratory distance of T47D cells was narrowed by butyrate treatment in a dose-dependent manner (Fig. 2B, C). The number of invasive T47D cell was reduced after butyrate treatment at the 2.0 and 5.0 mM doses (Fig. 2D). In summary, butyrate effectively suppresses the cell viability, migration and invasion of T47D cells in a dose-dependent manner.

### Butyrate inhibited BC tumor growth and promoted cuproptosis protein expression

**Fig. 3:**
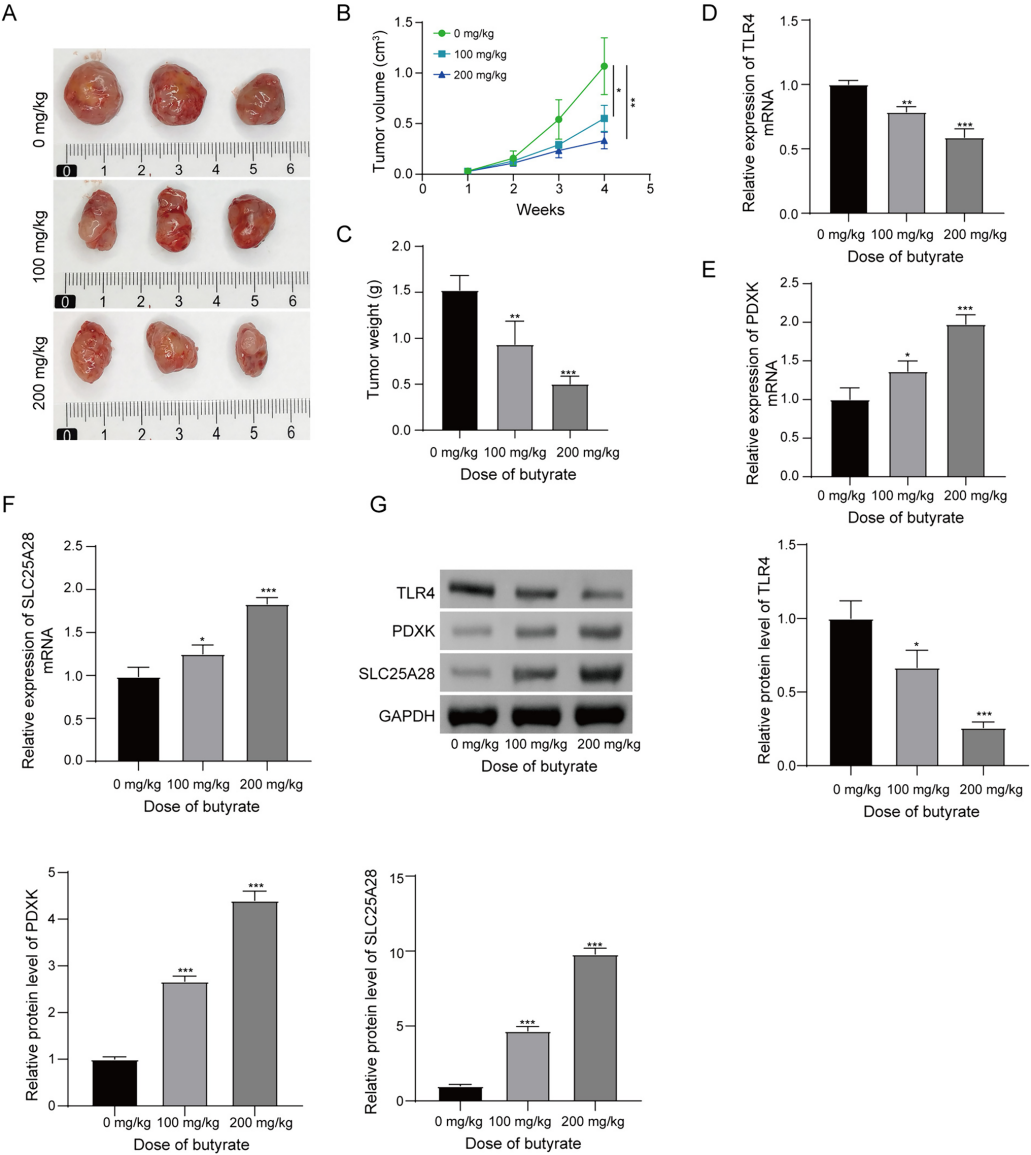
Butyrate impedes the growth of BC tumor. Tumor-bearing mice accepted with butyrate treatment at 0, 100 and 200 mg/kg doses. **A** The images of three tumors from each group were displayed. **B** Tumor volume was measured every week for 4 weeks. **C** Tumor weight was determined after four weeks of butyrate treatment. **D** The expression level of TLR4 mRNA in tumors was determined by RT-qPCR. **E** RT-qPCR revealed the expression level of PDXK mRNA. **F** SLC25A28 mRNA level in tumors was also measured by RT-qPCR assay. **G** The expression levels of TLR4, PDXK and SLC25A28 were measured by western blot assay. *P < 0.05, **P < 0.01, and ***P < 0.001. N = 3

The results of cellular experiments indicated that butyrate inhibited the malignant biological behaviors of T47D cells. Subsequently, we explored the effects of butyrate on the growth of BC tumors. Tumor mice were accepted with different doses (0, 100, and 200 mg/kg) of butyrate. Butyrate effectively inhibited BC tumor growth in a dose-dependent manner (Fig. 3A, B). The weight of tumors in the mice accepted with 100 and 200 mg/kg butyrate was lower than that in the mice accepted without butyrate (Fig. 3C). The expression of TLR4 mRNA (Fig. 3D) in tumors was downregulated by butyrate treatment in a dose-dependent manner. It has been reported that the serum level of copper in patients with BC is increased when compared with controls (Pavithra et al. 2015). As a mode of cell death caused by Cu accumulation, cuproptosis has been proved to inhibit cancer cell proliferation and tumor growth (Tong et al. 2022). Here, the expression levels of PDXK and SLC25A28 in tumors were detected. The expression of PDXK mRNA (Fig. 3E) and SLC25A28 mRNA (Fig. 3F) in tumors was upregulated after butyrate treatment at 100 and 200 mg/kg doses. When compared with the mice accepted without butyrate, the expression of PDXK and SLC25A28 proteins was increasing in the tumors which from the mice accepted with butyrate (Fig. 3G). Oppositely, the expression of TLR4 protein was downregulated in the tumors isolated from the mice accepted with butyrate (Fig. 3G). In summary, butyrate significantly inhibits the growth of BC tumor and facilitates the expression of PDXK and SLC25A28 in a dose-dependent manner.

**Fig. 4:**
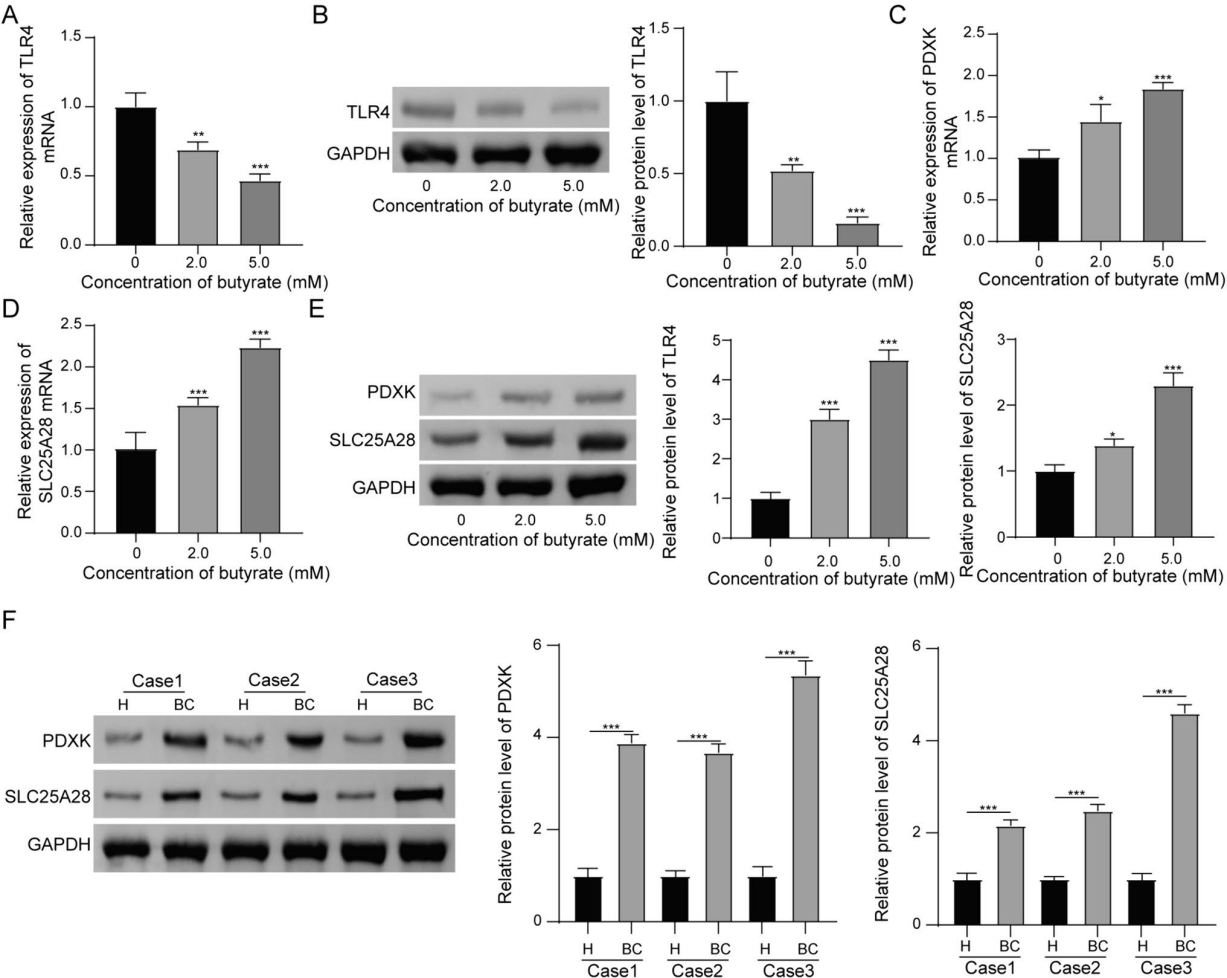
Butyrate inhibits TLR4 but facilitates PDXK and SLC25A28 expression. 0, 2.0, and 5.0 mM butyrate maintained with T47D cells for 48 h. **A** RT-qPCR revealed the expression level of TLR4 mRNA. **B** Western blot was carried out to analyze the level of TLR4 protein. **C**, **D** The mRNA expression levels of cuproptosis-related genes (PDXK and SLC25A28) were determined by RT-qPCR. **E** Western blot revealed the protein levels of PDXK and SLC25A28 in T47D cell. **F** 12 cases of paracancerous tissue and 12 cases of BC tumor specimen were obtained and used for detecting the expression of PDXK and SLC25A28. Three representative results were shown. *P < 0.05, **P < 0.01, and ***P < 0.001. N = 3

In cellular experiments, butyrate dose-dependently inhibited TLR4 mRNA (Fig. 4A) and TLR4 protein (Fig. 4B) expression in T47D cells. In addition, the expression of PDXK mRNA (Fig. 4C) and SLC25A28 mRNA (Fig. 4D) in T47D cells was increased by butyrate treatment in a dose-dependent manner. The results of western blot demonstrated that PDXK protein and SLC25A18 protein expression was also increased after the treatment of 2.0 and 5.0 mM butyrate (Fig. 4E). Moreover, decreased PDXK protein expression and decreased SLC25A28 protein expression were found in clinical BC tumor specimens (Fig. 4F). Based on the above data, butyrate may inhibit tumor growth through TLR4 and cuproptosis-associated proteins.

### *Butyrate suppressed the malignant biological behaviors of cells *via* TLR4 signalling*

**Fig. 5:**
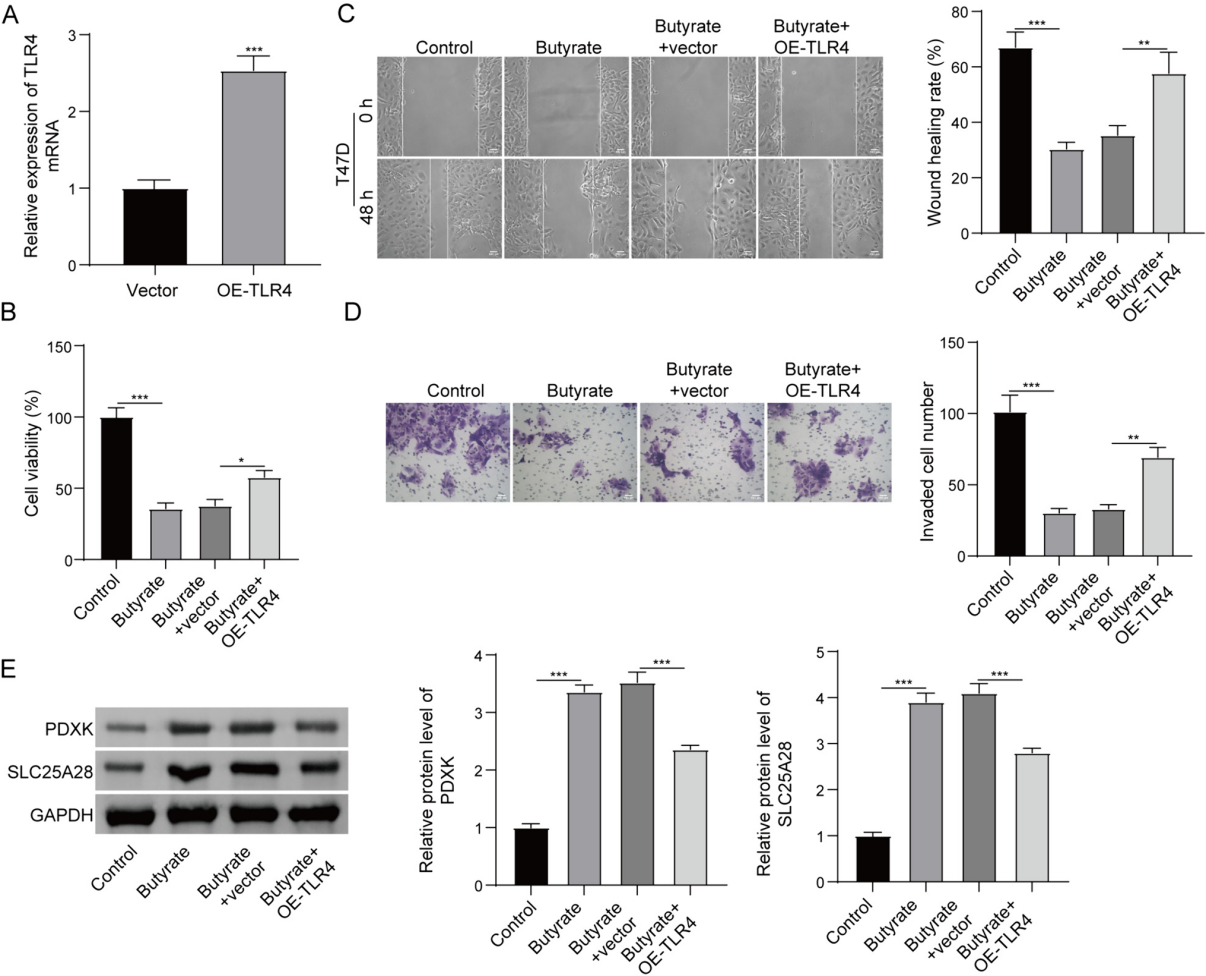
Butyrate inhibits TLR4 and the cell viability, migration and invasion of T47D cells by targeting PDXK and SLC25A28. **A** We transfected the plasmid expressing TLR4 and empty plasmid into T47D cells. RT-qPCR was utilized to detect the level of TLR4 mRNA. Moreover, T47D cells were treated with butyrate alone or in combination with TLR4 overexpression plasmid. **B** Cell viability was determined by CCK-8 assay. **C** Cell migration was determined by Wound healing assay. **D** Cell invasion was assessed by Transwell assay. **E** Western blot revealed the expression levels of PDXK and SLC25A28. *P < 0.05, **P < 0.01, and ***P < 0.001. N = 3

It was demonstrated that TLR4 is an oncogene in BC (Wang et al. 2018). Here, transfection of the plasmid expressing TLR4 led to a significantly increase in TLR4 expression in T47D cells (Fig. 5A). The effects of TLR4 on BC cell viability, migration and invasion, and its effect on SLC25A28 and PDXK expression were verified. Overexpression of TLR4 could enhance T47D cell viability (Supplementary Fig. 1A), migration (Supplementary Fig. 1B) and invasion (Supplementary Fig. 1C). Meantime, overexpression of TLR4 suppressed PDXK and SLC25A28 expression (Supplementary Fig. 1D). In order to ensure whether butyrate suppresses BC tumor growth by targeting TLR4-PDXK/SLC25A28 signalling pathway, T47D cells were transfected with the plasmid expressing TLR4 or empty plasmid, and simultaneously treated with 5.0 mM butyrate. Overexpression of TLR4 reversed butyrate-induced reduction to cell viability (Fig. 5B). The inhibition of butyrate to T47D cell migration (Fig. 5C) and invasion (Fig. 5D) was also rescued by increasing TLR4. Importantly, although the expression of PDXK and SLC25A28 was increased in T47D cells after butyrate treatment, TLR4 overexpression inhibited the expression of the above proteins (Fig. 5E). Overall, butyrate inhibits TLR4 expression and the cell viability, migration and invasion of T47D cells by promoting PDXK and SLC25A28 expression.

## Discussion

In the current study, we investigate the role of butyrate in development of BC, and the data indicate that butyrate inhibits the cell viability, migration and invasion of T47D and impedes the growth of BC tumors. We further discussed the underlying mechanism of butyrate. TLR4, a known downstream target of butyrate, is highly expressed in BC tumors. Cuproptosis-associated genes (PDXK and SLC25A28) are lowly expressed in BC tumors. In BC cell lines and tumors, butyrate suppresses TLR4 expression, while facilitates PDXK and SLC25A28 expression.

A large number of evidence point to an important association between the risk and progression of cancers and the gut microbiota. Gut microbiota can impact circulating estrogen levels, which is associated with the development of some cancers like ovarian cancer and BC (Łaniewski 2020). In addition, gut microbiota also involves the development of cancer by interacting with the immune system and releasing various metabolites into the blood, including enterolignans, cadaverine, and SCFA (Matson et al. 2021; Li et al. 2019; Chen et al. 2021). SCFAs are characterized by an aliphatic tail ranging from one to six carbons, which common types being acetate, butyrate, isobutyrate, propionate, hexanoate, and valerate. Among these SCFAs, acetate, propionate, and butyrate have been shown to inhibit cancer development (Liu et al. 2021). Abnormal levels of acetate, propionate and butyrate in faecal samples have been associated with inflammatory parameters in patients with pancreatic cancer, lung cancer, ovarian cancer or BC (Ubachs et al. 2021). In the current study, abnormal production of acetate, propionate and butyrate was identified in the faecal samples of BC patients. The levels of acetate, propionate and butyrate are lower in the faecal samples of BC patients than that in control samples. In addition, quite a few recent studies have demonstrated the anti-cancer effects of SCFAs in BC. For instance, propionate and butyrate could inhibit the proliferation of BC cell line MCF7 and arrest cell cycle in the G1 phase (Semaan et al. 2020). However, there is limited research on the role and regulatory mechanisms of butyrate in BC development. Our findings prove that butyrate effectively suppresses the cell viability, migration and invasion of BC cell line T47D, as well as inhibiting experimental tumor growth.

Furthermore, our findings indicate that butyrate inhibits TLR4 expression in a dose-dependent manner, potentially impacting its biological function. TLR4, a member of the pattern recognition receptor TLRs family, plays a crucial role in cancer development by regulating the production of chemokines and pro-inflammatory cytokines (Chen et al. 2018). Wang et al. demonstrated that resistin could accelerate BC development through induction of stemness properties and mesenchymal phenotypes in BC cells by activating TLR4/NF-κB/STAT3 signalling pathway (Wang et al. 2018). Li et al. (2017) indicated that TLR4 is overexpressed in BC tumors, and activated TLR4 promotes the migration of BC cells by regulating AKT/GSK3β/β-catenin signalling pathway. Consistent with the study of Li et al., high expression of TLR4 in BC tumors is also find in our present study. The expression of TLR4 in BC cell lines is higher than that in control cell line. Importantly, our findings show the inhibition of butyrate to TLR4 expression in a dose-dependent manner. TLR4 is a downstream target of butyrate, and this point has been confirmed by several studies (Sun et al. 2021; Seth et al. 2018). Nevertheless, this study is the first to report on the role of TLR4 in the suppression of butyrate on BC.

In recent years, several lines of evidence have implicated that cuproptosis-related genes, including SLC31A1, DLAT, and ATP7B, could serve as potential diagnostic and therapeutic targets for BC (Li et al. 2022a, b). Here, our data demonstrate low expression levels of cuproptosis-related genes PDXK and SLC25A28 in BC tumors. Previous studies have linked PDXK to the progression of pancreatic ductal adenocarcinoma (PDAC). Circular RNA FOXK2 aggravates PDAC tumor growth and metastasis by affecting PDXK and NUF2 expression through interaction with RNA binding protein YBX1 and hnRNPK (Wong et al. 2020). The role of SLC25A28 in cancer development remains unexplored. Importantly, in our present study, butyrate treatment upregulates the expression of PDXK and SLC25A28 in cellular and animal experiments, suggesting a potential inhibitory effect of butyrate on BC development through the regulation of cuproptosis or cuproptosis-related genes. The relationship between TLR4 and PDXK/SLC25A28 has been previously reported. To explore whether the butyrate-induced upregulation of PDXK and SLC25A28 is associated with TLR4, we restored TLR4 expression in butyrate-treated T47D. Proof by facts, overexpression of TLR4 rescues the butyrate-induced upregulation of PDXK and SLC25A28, as well as the inhibition of butyrate to the cell viability, migration and invasion of T47D cells.

## Conclusion

Butyrate, a representative SCFA, inhibits the cell viability, migration and invasion of BC cells and the growth of BC tumors by increasing PDXK and SLC25A28 via suppression of TLR4. Our research, for the first time, indicates the correlation among butyrate, TLR4 and cuproptosis-related PDXK/SLC25A28 in the development of BC. Our study may contribute to find novel therapeutic target for BC. Nevertheless, in this research there still exist some limitations. The mechanism that butyrate suppresses TLR4 still not known. SCFAs are thought to exert their functions via protein epigenetic modification like acetylation. Butyrate adopts epigenetic approaches that mediated acetylation of histones to promote the expression of target genes (Rangan and Mondino 2022; Zhu et al. 2023; Lu et al. 2022b). In following studies, the epigenetic regulation of SCFAs to TLR4 and other targets will be one of our research focuses.

### Supplementary Information

Below is the link to the electronic supplementary material.

Supplementary Fig. 1. TLR4 facilitated BC cell viability, migration and invasion, but inhibited PDXK and SLC25A28 expression in T47D cells. T47D cells were transfected with the vector expressing TLR4 or empty vector. Subsequently, (A) cell viability was determined by CCK-8 assay, (B) cell migration was detected by Wound healing assay, and (C) cell invasion was assessed by Transwell assay. (D) The expression of PDXK and SLC25A28 was detected using western blot. *P < 0.05, **P < 0.01, and ***P < 0.001. N = 3 (DOCX 15 KB)

Supplementary file2 (TIF 8798 KB)

## Data Availability

All data generated or analyzed are included in this article. Further inquiries can be directed to the corresponding author.

## Abbreviations

BC: Breast cancer
BCFAs: Branched-chain fatty acids
PDAC: Pancreatic ductal adenocarcinoma
PDXK: Pyridoxal kinase
SCFA: Short-chain fatty acids
SLC31A1: Solute carrier family 31 member 1
SLC25A28: Solute carrier family 25 member 28
TLR4: Toll like receptor 4
