# Supplementary material for: Butyrate inhibits the malignant biological behaviors of breast cancer cells by facilitating cuproptosis-associated gene expression
Source: J Cancer Res Clin Oncol. 2024 Jun 4;150(6):287. doi: 10.1007/s00432-024-05807-1 (PMC11150186; doi:10.1007/s00432-024-05807-1)
Supplement: Supplementary file 1 — Supplementary Fig. 1. TLR4 facilitated BC cell viability, migration and invasion, but inhibited PDXK and SLC25A28 expression in T47D cells. T47D cells were transfected with the vector expressing TLR4 or empty vector. Subsequently, (A) cell viability was determined by CCK-8 assay, (B) cell migration was detected by Wound healing assay, and (C) cell invasion was assessed by Transwell assay. (D) The expression of PDXK and SLC25A28 was detected using western blot. *P < 0.05, **P < 0.01, and ***P < 0.001. N = 3 (DOCX 15 KB) [file 432_2024_5807_MOESM1_ESM.docx]

Table1 Clinicopathological characteristics of breast cancer patients

| Sample ID | Age | Gender | Pathological type | Histological grade | Molecular subtype |
| --- | --- | --- | --- | --- | --- |
| 1 | 39 | Female | IDC | II | Luminal A |
| 2 | 42 | Female | IDC | III | HER2-enriched |
| 3 | 61 | Female | IDC | III | HER2-enriched |
| 4 | 42 | Female | IDC | III | HER2-enriched |
| 5 | 64 | Female | IDC | III | HER2-enriched |
| 6 | 53 | Female | IDC | III | TNBC |
| 7 | 47 | Female | IDC | III | HER2-enriched |
| 8 | 59 | Female | IDC | III | HER2-enriched |
| 9 | 45 | Female | IDC | II | Luminal A |
| 10 | 51 | Female | IDC | III | HER2-enriched |
| 11 | 40 | Female | IDC | III | TNBC |
| 12 | 56 | Female | IDC | III | TNBC |

Abbreviations: IDC, invasive ductal breast cancer; HER2, human epidermal growth factor receptor 2; TNBC, triple negative breast cancer.
