# Supplementary figures and images for: Butyrate inhibits the malignant biological behaviors of breast cancer cells by facilitating cuproptosis-associated gene expression
Source: J Cancer Res Clin Oncol. 2024 Jun 4;150(6):287. doi: 10.1007/s00432-024-05807-1 (PMC11150186; doi:10.1007/s00432-024-05807-1)

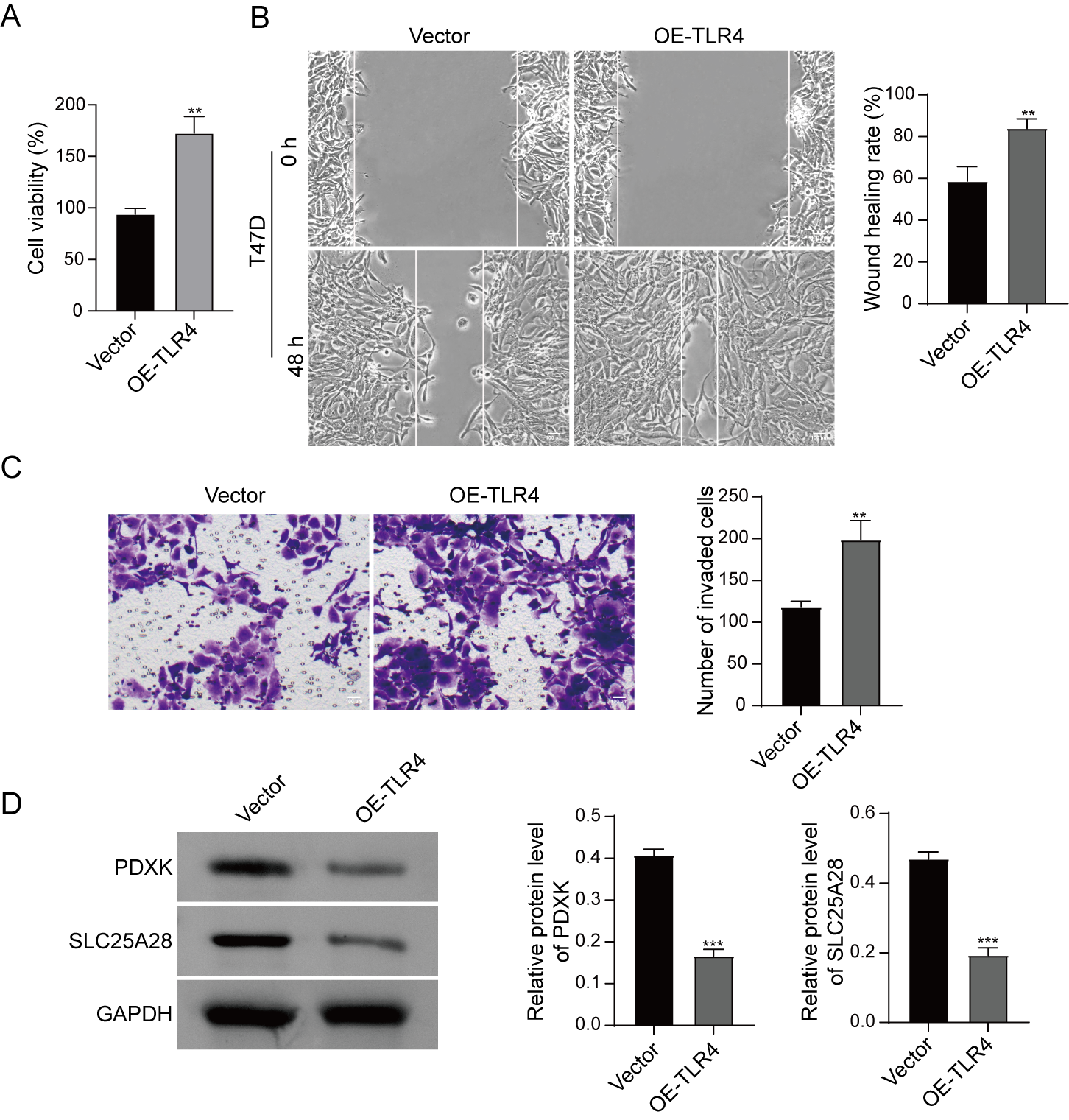

Supplement: Supplementary file 2 — Supplementary file2 (TIF 8798 KB) [file 432_2024_5807_MOESM2_ESM.tif]
